# Case of Necrosis of Portions of Maxillary Bones, Caused by Excessive Salivation

**Published:** 1871-07

**Authors:** S. P. Cutler


### ARTICLE II.

### *Case of Necrosis of Portions of Maxillary Bones, caused by Excessive Salivation.*

By S. P. Cutler, M. D., D. D. S.

Read before the New Orleans Dental Association.

A beautiful little girl five years old, called for the purpose of having some teeth removed that were loose from salivation. Her father had taken her to an occulist to have the remaining eye treated, one having gone out from the disease; the occulist thinking the teeth had some connection with the eye thought they had better be taken out.

The father said the child had pneumonia, that the physician had given her three 5 grain doses of calomel which operated favorably; but in the course of three or four days profuse salivation set in, attended with severe swelling of the whole mouth, the inflammation extending to the right eye causing the ball to finally burst and discharge the humors. This happened about three months before I saw the case. The inflammation had subsided entirely, only what remained in consequence of the loose pieces of bone. The left eye was becoming slightly milky in appearance, owing no doubt to the irritation from the diseased bones of the mouth.

On examination, I found the lower molars and cuspid of the right side loose, and evidently portions of bone. I at once took hold of the loose tooth with my thumb and finger, and removed the two molars and alveolus entire and some distance below without any force, the tooth and bone together, the cuspid dropping into the mouth on removal of the other teeth and bones. After removal of the bone I noticed the crowns of the two bicuspids perfectly bare and loose, which the child pushed out of their beds with her tongue, the bone having exfoliated down to a line with the lower surface of the gums. These bicuspid crowns and their pulps were perfectly natural and healthy, which I preserved for microscopical examination. The pulp extended across their base, plump and sound and slightly convex.

The two upper molars on the same side were in a similar condition, which I removed in the same way without the aid of any instrument at all. The bone removed was more extensive than the lower including the crowns of the two bicuspids; the anterior one being visible across the pulps and a portion of the anterior surface of the crown. The second bicuspid being entirely hid by the bone coming away higher up including the floor of the pulp's cavity, a concavo convex matrix, corresponding to form of pulp, the outer portion opposite running up and including portion of as mala.

The pulps of these two bicuspids were destroyed, as they were included in the necrosed portion of bone. It will be recollected that the crowns of the permanent bicuspids are situated immediately under the deciduous molars between the fangs with bone interposing between the deciduous fangs; only in some cases there may be partial contact with fangs, which was the case with one of the above—the second bicuspid with posterior fang of second molar.

On the left side, the disease was limited to the anterior lower molar and cuspid with investing alveolus, which I removed with a small forcep without using any force scarcely at all, the cuspid having previously fallen out; the alveolus formerly occupied by this tooth coming away with the molar. The upper jaw on the left side, was healthy as well as the balance of the mouth.

There was scarcely any hemorrhage at all, the soft parts falling into vacant spaces at once, as though nature had been waiting this event in order to effect a speedy cure. The little patient did not shed a tear, but seemed to feel decidedly relieved and gratified. I did not hear from the case afterward, though I have not the least doubt but that she speedily recovered from all her troubles.

These bones were very offensive indeed, as is the case always in necrosed bone, especially if resulting from the effects of mercury.

All the diseased bone, the sequestrum, came away as they were quite loose in their places and the surrounding parts assumed a healthy appearance.

I believe the surrounding bone to have undergone absorption and healing so far as it possibly could with the loose pieces held in their places mechanically by the soft parts; and I have no doubt but that a new periosteum was already formed under the sequestered bone, ready to unite with the gum and mucous covering as soon as the removal of pieces of bone was accomplished.

This little patient had a fine, naturally robust constitution, not a speck of decay on any one of her teeth, and will no doubt entirely recover from the terrible effects of this uncalled for, and unnecessary mercurialization, with only the deformities which are irremediable; those of the mouth will not be noticeable to any extent.

Here was clearly a case, either of accident or malpractice neither of which is excusable, as it is the duty of practitioners of medicine to narrowly watch the effects of their prescriptions, especially when mercury is used. In this case the doses of mercury were evidently too heroically administered, as children of that age are not subject to bilious engorgements sufficiently to indicate the treatment often resorted to, the disease being that of the lungs and not per se that of the liver. I do not approve of censuring a prac titioner of medicine, when he does the very best according to his judgement, though oftentimes they are too careless and negligent when life and death are in their keeping.

The crowns and pulps of the four bicuspids occupy a crypt or cavity in solid bone situated between the fangs of the deciduous molars, or in other words a bony sack which may be separated from the balance of the bone without much trouble, though connected with it by usual bony union, only this portion is lined by the outer original sack containing the crown and pulp. This space formerly occupied by the sack, is left around the crowns in this case, this membrane having sloughed away, even from the two lower in the right side already described, which were in contact with the lower portion of the crypt by vital union which was easily separated, leaving the pulps plump and healthy in appearance. The condition between the pulps and the existing sack which lines the bony cavity already alluded to, is fleshy receiving the fasciculi of nerves and the blood vessels which supply the tooth, with probably some other union with the membraneous sack. There are a number of small holes in the bony crypt for arteries and veins to pass in for nutrition. This crypt is nearly double the size in depth of the crown, the pulp's investing membrane filling the additional space not taken up by the crown.

There is apparently no connection between the interior circulation and neural arrangement, between the deciduous and permanent teeth, each apparently existing independently of each other. On looking into the pulp's fossa of the bicuspid in situ, two deep indentations are conspicuous, corresponding to the two pulps. This pulp fossa is shallow not claiming the distinction of a cavity as yet. These crowns are completed to the first portion of the future neck, where the enamel bevels off to a thin edge, within dentine and without enamel; the thin edge of enamel forming a regular bevel in the two coming away with the bone apparently from incompleteness, the others not so much so.

It must be borne in mind that the dentine and cement constituting the neck and fang, have to be yet developed, the bone, even when not of sufficient depth to admit of alveolus, still absorbs and makes way for the tooth which begins to come up at the time that the fang begins to develop down into the depths of the bone. The tooth as it comes up brings up with it in its development the alveolus or a portion of it, which is a double developemental process.

I made a section through the centre of one containing the pulp which I had treated with alcohol to harden it.

The dentine is apparently better developed or better perfected than enamel, and much harder, which is not the case after eruption. The enamel rods, as they have been termed, are all fully developed and perfect in organic structure with scarcely any earthy salts, except the portion in contact with dentine. The rods in this region contained lime deposits, though not apparently fully organized, seeming more porous, and not that peculiar crystalloid structure witnessed in fully developed teeth.

The enamel structure may be filled with soft lime deposit undergoing condensation to a certain extent; the lime gradually displacing the colloid; matter somewhat similar to the process of petrifaction of organic structures, the chief difference being that of chemico-vital on the one hand, and chemical or mineral on the the other; the one taking place in living structures, the other in structures once living.

These comparisons are not altogether fanciful or imaginary speculations. Nobody at the present time knows exactly how this process of enamel developement takes place, we are still groping in the dark as on many other kindred subjects.

One thing we are certain of in this relation, that is, that the development and perfection of enamel is a very slow process requiring a great number of years for its complete perfection, ranging from the time of the first commencement of the formation of dentine until sometime after the eruption of the tooth.

The pulps of the bicuspids at this period are in the form of double convex lines of high powers, one convex surface looking down from crown, the other looking into the tooth towards grinding surface, the pulp being oval, transversely across the tooth, seeming to unite with the interior of the organ so far as there is any interior· The border of the enamel is not apparently in connexion with pulp, there being a small space all around the pulp where the enamel stand a little away from this organ which is quite visible to the naked eye. The border of enamel, it will be recollected, is the last formed, and consequently this portion is is least dense, causing the limited margin spoken of the effect of shrinkage.

I succeeded in sawing through the tooth, enamel, dentine and pulp, and retained them intact to some extent, sufficient to show the union by fibrillæ, though not all the way, only a part. At one point the fibrillæ openings through, pulp membrane are very distinct. The nerve fibrils are not as distinct as I have seen them in fully developed teeth, nor as distinct as I could wish for definite date at this stage, but showing the characteristic structures of pulp substance so often referred to by myself in former papers.

The proportion of crown to pulp, is about as 2 to 1, or 2 to 3, hence the proportion of pulp to tooth is much greater at this period than at maturity. The tubular structure of the dentine is about the same as in fully developed deciduous teeth so far as the dentine is developed. The dentine is formed from the inter-dental, if the term is allowable, circulation, the enamel and cement from extra-dental circulation, or from vessels not in any way, so far as we know, connected with the interior or inter-dental or *papal* circulation, unless by anastomosis. After the crown of the tooth is formed, this crown then has a stadium prodromerum, or a stage of probation, during which time there is not much change, not until growth is again commenced which is the eruptive growth, after which the stage is completed, and the fully formed tooth is again stationary, comparatively speaking until advancing age, then again another process is set up which continues to the end. This is a down grade movement.

The importance of keeping the mouths of children in a normal healthy condition during the entire time of dentition, or from birth up to puberty and manhood, cannot be too highly appreciated by the profession and the people at large. Too much care and vigilance cannot be exercised in this direction. Any decayed, diseased and aching deciduous teeth causing congestion and inflammation of the bone enclosing the permanent successors, necessarily disturbs more or less the health of the permanent, so much so as to greatly interfere with the ossifying process, in consequence of which the permanent teeth decay early after eruption from this very cause. Any disease seriously affecting the health of the mouth, produces this effect. The exanthemata diseases at certain ages and *syphilis constitutionalis* are all disturbing causes, and affect more or less the health of the new tooth.

In the above case, the cause of the extensive necrosis was the mercurial inflammation first attacking the oral glands, primarily the mucous membrane; secondly and thirdly, the periosteum covering the alveolus and lining the sockets and fangs of the teeth, cutting off osseous nutrition from the bone surface first, causing death there, subsequently passing across and through the bone to meet the same effect passing in from the opposite side. The failure of nutrition of the surface of the bone from inflammation or any other cause, for an indefinite period, necessarily causes death to strike into the bone, or in other words starvation of the bone ending in death and sloughing, and a dead line being established and vital connexion severed by what is understood as bone absorption, right at the dead line by the live bone beneath, the result of chemical vital action, which must of necessity be influenced by the presence of some organic acid, though no such effect is discoverable in the sequestrum, the animal matter of the cancellæ being all gone from putrefactive decomposition. This mercurial inflammation could and should have been overcome by porper treatment, in time to save the life of the bone, as it was not the work of a day or of even three or four, but of six or eight or more, before death began at the surface of the bone. I

do not speak definitely as to time only by approximation.

I well remember thirty odd years ago, when I was practicing medicine in a malarious district in Tennessee, that it was a universal custom to salivate in almost all cases of of fever of whatever character; and similar cases to the one mentioned in this paper were not unfrequent in children, some even sweeping away the entire alveolus of the lower jaw, teeth and all. When a tooth pulp is destroyed there is a dead line somewhere, established probably at the junction of the cement and dentine, in some cases still unlike death of a portion of bone anywhere in the system, no sequestrum is formed or any separation of the living from the dead, as in the case of bone, takes place. The only effort made by the vital forces is to throw off the tooth bodily from the jawbone, which she is incompetent to do only to a partial extent. From these facts we arrive at the conclusion that teeth are something different from ordinary bones, even the cement. Teeth are highly endowed tissue, while, bone on the contrary is very low in the vital scale. Injured or diseased bones, are either restored or the diseased portion separated, not so with teeth as before stated.

Tomes in his work, chapter fifth, gives the best histological treatise on dentition extant, embodying the labors and observation of Mr. Goodsir and others. He speaks of the fact that the enamel is not completed until after eruption, but does not give the exact condition and hardness of the enamel at any particular period of development only in a general way.

---

